# Genome-Wide Transcriptional Roles of KSHV Viral Interferon Regulatory Factors in Oral Epithelial Cells

**DOI:** 10.3390/v16060846

**Published:** 2024-05-25

**Authors:** Seung Jin Jang, Natalie Atyeo, Mario Mietzsch, Min Y. Chae, Robert McKenna, Zsolt Toth, Bernadett Papp

**Affiliations:** 1 Department of Oral Biology, University of Florida College of Dentistry, 1395 Center Drive, Gainesville, FL 32610, USA; 2 Department of Biochemistry and Molecular Biology, University of Florida College of Medicine, 1200 Newell Drive, Gainesville, FL 32610, USA; 3 UF Genetics Institute, Gainesville, FL 32610, USA; 4 UF Health Cancer Center, Gainesville, FL 32610, USA; 5 UF Center for Orphaned Autoimmune Disorders, Gainesville, FL 32610, USA; 6 UF Informatics Institute, Gainesville, FL 32610, USA

**Keywords:** KSHV, vIRF, gene regulation, oral epithelial cells, gammaherpesviruses, IRF, genomics, EDC genes

## Abstract

The viral interferon regulatory factors (vIRFs) of KSHV are known to dysregulate cell signaling pathways to promote viral oncogenesis and to block antiviral immune responses to facilitate infection. However, it remains unknown to what extent each vIRF plays a role in gene regulation. To address this, we performed a comparative analysis of the protein structures and gene regulation of the four vIRFs. Our structure prediction analysis revealed that despite their low amino acid sequence similarity, vIRFs exhibit high structural homology in both their DNA-binding domain (DBD) and IRF association domain. However, despite this shared structural homology, we demonstrate that each vIRF regulates a distinct set of KSHV gene promoters and human genes in epithelial cells. We also found that the DBD of vIRF1 is essential in regulating the expression of its target genes. We propose that the structurally similar vIRFs evolved to possess specialized transcriptional functions to regulate specific genes.

## 1. Introduction

Kaposi’s sarcoma-associated herpesvirus (KSHV) is an oncogenic DNA virus that can cause multiple cancers in immunocompromised individuals, including Kaposi’s sarcoma (KS), primary effusion lymphoma (PEL) and a subset of multicentric Castleman’s disease (MCD) [1,2,3]. Epidemiological and clinical studies indicate that the oral cavity plays a particularly important role in the transmission and pathogenesis of KSHV infection [4,5]. The infection of oral endothelial or mesenchymal stem cells can result in oral KS, which is a frequent cancer in patients with acquired immunodeficiency syndrome (AIDS) [6]. Testing the survival of a cohort of KS patients with AIDS revealed that patients with oral KS had a greater than three-fold higher death rate than those with cutaneous KS [7]. It has also been observed that KSHV is shed in the oropharynx and can be transmitted via the saliva [8,9]. As KSHV can replicate in human gingival epithelial (HGEP) cells following de novo infection, it has been suggested to be one of the potential sources of virions in the saliva [10,11]. Hence, the oral cavity is a biologically relevant site for KSHV infection. 

To establish a chronic infection, KSHV has evolved several strategies to evade the host immune response triggered by viral infection [12]. It is unique among human viruses as it expresses four viral homologs of the cellular interferon regulatory factors (IRFs) [13]. While KSHV vIRF1, vIRF2 and vIRF4 are mainly expressed as lytic viral genes, vIRF3 was originally identified as a latent viral factor expressed mostly in PEL cells and MCD tumors. However, recent studies showed that vIRF3 can also be induced as a lytic gene [14,15,16,17]. Each vIRF can inhibit interferon (IFN) signaling or the expression of IFN-stimulated genes by different mechanisms [17,18,19]—for example, by blocking the function of cellular IRFs, cyclic GMP-AMP synthase (cGAS) or Toll-like receptor (TLR) signaling pathways or nuclear factor-KappaB (NF-kB)-mediated gene transcription [20,21]. It was shown that vIRF1 and vIRF3 can directly repress type I IFN promoters through blocking the interaction between IRF3 and its transcriptional activators, such as the histone acetyltransferases CBP/p300 [22].

The N-termini of vIRFs are homologous to the DNA-binding domains of cellular IRFs, although they lack several of the tryptophan residues that are essential for the DNA-binding activity of cellular IRFs [23]. However, the crystal structures of the putative DNA-binding domains of vIRF1 and vIRF2 with DNA revealed that there are specific arginine amino acids in the N-termini of vIRF1 and vIRF2 that can mediate a direct interaction with DNA [23,24]. Interestingly, direct DNA binding can only be observed with the N-terminal vIRF fragments and not with the full length of vIRFs using purified proteins in vitro [23,24]. In contrast, in vivo, full-length vIRF1 was shown to be able to directly bind to the promoter of the KSHV gene K3 [25]. It is hypothesized that the C-termini of vIRFs inhibit or interfere with their DNA-binding activity, and this inhibition is relieved by the binding of vIRFs to other proteins in vivo [23]. These findings suggest that the DNA-binding mechanisms of cellular and viral IRFs are likely different.

KSHV has a large repertoire of viral factors that can mediate immune evasion in the oral cavity, allowing the virus to subvert the first line of defense in the host to establish a chronic infection. In this regard, vIRFs can potentially contribute to promoting KSHV replication in oral epithelial cells. However, most of the research on vIRFs has been performed in endothelial cells and B cell lymphoma cells and focused on their inhibitory roles in different immune response pathways and how they can be involved in oncogenic pathways [13]. Many of these functions were linked to the cytoplasmic functions of vIRFs [20]. In contrast, it is still unclear to what extent (i) vIRFs modulate the host and viral transcriptomes to control KSHV infection in oral epithelial cells and (ii) the importance of the DNA binding of vIRFs in vIRF-mediated gene regulation. To comprehensively address these questions, we performed a comparative structural analysis of the vIRFs, a viral promoter luciferase reporter screen to identify the KSHV promoters that can be controlled by vIRFs and a host transcriptome analysis to determine the host genes regulated by the vIRFs. Furthermore, we provide evidence for the DNA-binding function of vIRF1 being required for its gene transcription regulatory function in oral epithelial cells genome-wide.

## 2. Materials and Methods

### 2.1. Structure Prediction and Comparison

For disorder predictions, the PONDR_fit application (version 6.02) was used [26]. Similarly, the primary amino sequence was used to generate structure predictions with AlphaFold v2.3.1 [27]. The top-ranked model was utilized for all vIRF proteins. For the graphical representations of the models, PyMol and Chimera were used [28,29]. For the structural comparison, the individual coordinate models were superposed in Coot [30]. The structural identity was defined as the percentage of aligned amino acids where the distance of the Cα atoms was ≤2 Å. 

### 2.2. Cell Lines

Human gingival epithelial cells (HGEP) were purchased from CellnTec and maintained in CnT-prime epithelial proliferation medium (CnT-PR). hTERT-immortalized gingival keratinocytes (TIGK) were obtained from ATCC and maintained in dermal cell basal medium supplemented with the keratinocyte growth kit (ATCC). HEK293T cells (ATCC) were cultured in DMEM medium containing 10% FBS, 100 U/mL penicillin and 10 μg/mL streptomycin (P/S).

### 2.3. Antibodies, Plasmids and DNA Transfection

The following primary antibodies were used in the study: anti-FLAG (#1804, Sigma, St. Louis, MO, USA), anti-Tubulin (Sigma, T5326), anti-ORF45 (sc-53883, SCBT Dallas, TX, USA), anti-K2 (#251352, Abbiotec, Escondido, CA, USA), anti-EGR1 (NBP1-78775, Novus Biologicals, Centennial, CO, USA), anti-H3K4me3 (#39159, Active Motif, Carlsbad, CA, USA) and anti-H3K27ac (Active Motif, #39133). The lentiviral expression vector pCDH-CMV-MCS-EF1-puro was used for the expression of N-terminally 3xFLAG epitope-tagged vIRFs and the 3xFLAG-vIRF1 mutants. In the DNA-binding mutant of vIRF1, the arginine residues 163 and 172 were changed to alanine. The USP7-binding mutant of vIRF1 was generated by deleting the coding sequence of the amino acid residues 47 to 50 (ΔEGPS) [31]. The ORF57 promoter and its deletion mutants were cloned into the promoterless pGL4.15 luciferase reporter plasmid (Promega, Madison, WI, USA), while the RTA response element (RRE) sequence and a part of the ORF57 promoter were cloned into the luciferase reporter plasmid pGL4.27 possessing a minimal promoter (Promega). The 450-bp ORF57 promoter corresponds to the KSHV genomic DNA sequence (GenBank: GQ994935) between nt 81,371 and 81,821. The RRE in the ORF57 promoter corresponds to the KSHV genomic sequence between nt 81,715 and 81,777, and it was cloned into the Xho I site of the pGL4.27 vector. We used In-Fusion cloning to insert the PCR-amplified DNA fragments into their target plasmids (TaKaRa, San Jose, CA, USA). The construction of the KSHV promoter luciferase reporter library is described elsewhere [32]. The plasmid transfection of HEK293T cells was performed with polyethyleneimine (PEI 25K) from Polysciences (Warrington, PA, USA).

### 2.4. Viruses and Viral Infections

For KSHV infection, the BAC16 clone of KSHV was used, which was produced from the iSLKBAC16 cell line [33], and the infection was performed as described previously [34]. Lentiviruses expressing vIRFs were produced in HEK293T cells by co-transfecting the cells with the lenti-vIRF vectors along with three packaging plasmids. Three days after transfection, the cell culture media were collected, filtered and ultracentrifuged. The lentivirus pellets were resuspended in oral epithelial cell culture medium. The lentiviral transduction of HGEP and TIGK cells was performed by spinoculation in the presence of 8 μg/mL Polybrene. Six hours after the lentiviral transduction, the medium on cells was replaced with fresh medium containing P/S and the cells were harvested at the indicated time points.

### 2.5. Luciferase Reporter Assay

For this assay 80,000 HEK293T cells were seeded per well in 24-well dishes the day before transfection. Each well was co-transfected with 100 ng of a luciferase reporter plasmid and 400 ng of a vIRF expression vector or a control vector using PEI. At 36–48 h post-transfection, the cells in each well were lysed with 200 μL of lysis buffer (0.5% Triton X-100 in 1X DPBS). After centrifugation, 20 μL of cleared lysates were mixed with 20 μL of luciferase substrate (E6120, Promega) and the luciferase activity was measured by the Promega GloMax-Multi Detection System. All luciferase assays were performed in triplicate. The results of the luciferase assays were based on three independent experiments. The fold change in luciferase activity was calculated as the ratio of the luciferase activity in cells transfected with vIRFs relative to that in vector-transfected cells. For the significance test, we applied Student’s *t* test, and *p* ≤ 0.05 was considered significant.

### 2.6. RNA Isolation and RT-qPCR Analysis

Total RNA was isolated from cells using TRIzol reagent (Invitrogen) following the manufacturer’s instructions. The cDNA synthesis was performed with 1 μg of DNase I-treated total RNA using the iScript cDNA Synthesis Kit (Bio-Rad, Hercules, CA, USA). The RT-qPCR primers are listed in 5′ to 3′ orientation in Table 1. The qPCR was run on a CFX96 real-time PCR machine (Bio-Rad) using SYBR Green supermix (Bio-Rad). The relative gene expression changes were calculated by the ΔΔCt method, in which the expression of the housekeeping gene 18S was used for normalization. The gene expression graphs were based on three biological replicates. For the significance test, we used the one-tailed Student’s *t* test, and *p* ≤ 0.05 was considered significant.

### 2.7. Chromatin Immunoprecipitation Assay (ChIP)

Chromatin was prepared from 1.5 × 10^6^ HGEP cells that were transduced with lenti-GFP, lenti-3xFLAG-vIRF1 or lenti-3xFLAG-vIRF1/DBDm for 2 days. The chromatin preparation and the ChIP assay were performed as described previously [35]. We used 2 μg of chromatin and 0.5 μg of antibody per ChIP. The ChIP graphs are based on 3 independent ChIP experiments, and they show the percentage of the immunoprecipitated DNA relative to the input DNA for the given promoter or genomic region. The sequences of the primers used in the ChIP-qPCR are shown in Table 1.

### 2.8. RNA-Seq Analysis

Total RNA was extracted from HGEP cells using an RNeasy kit 48 h after lentivirus transduction. Strand-specific RNA-seq libraries were prepared from 3 biological replicates of lentiviral transduced HGEP cells by the Illumina-compatible NEBNext Ultra II Library Prep Kit and the resulting pair-end 150 (PE150) libraries were sequenced by Novogene. The sequence alignments to human transcripts were carried out by Novogene’s bioinformatics service. The differential expression analysis was performed by the DESeq2 Bioconductor R package, and significantly regulated genes were identified with a cut-off of at least a 1.5-fold change and padj < 0.05. The volcano plot diagrams depict the fold change in the gene expression levels at the *x*-axis, and the *y*-axis shows the significance of the differential gene expression. The Venn diagram of the gene expression analysis represents the number of differentially expressed genes in each group and the overlaps between each group as indicated. The functional annotations, including the gene ontology biological process (BP) and cellular component (CC) analysis of the list of gene sets, were performed on the Database for Annotation, Visualization and Integrated Discovery (DAVID) analysis platform. The RNA-seq datasets will be made available in the NCBI Gene Expression Omnibus (GEO) database with public accession numbers upon publication.

## 3. Results

### 3.1. Comparison of the Putative Tertiary Structures of vIRFs

**Figure 1:**
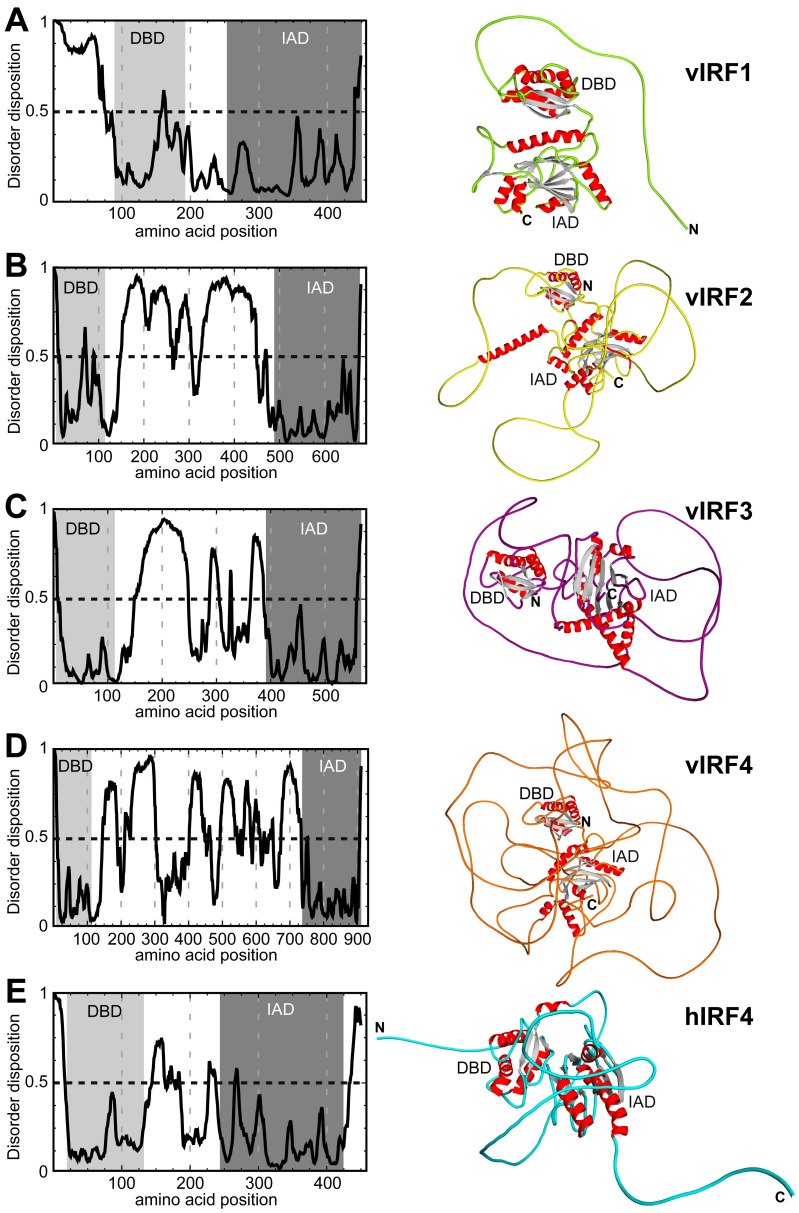
Structure prediction of KSHV vIRF1 (**A**), vIRF2 (**B**), vIRF3 (**C**), vIRF4 (**D**) and hIRF4 (**E**). Shown on the left are graphs of the predicted disorder disposition (*Y*-axis) for each of the vIRFs and hIRF4 along their amino acid sequences, calculated by PONDR_fit [26]. Regions with a disorder probability higher than 0.5 are predicted to be disordered. The light gray shaded area indicates the DNA-binding domain (DBD) and the dark gray shaded area displays the IRF association domain (IAD). On the right, the top-ranked AlphaFold structure predictions of the vIRFs and hIRF4 are shown as ribbon diagrams [27]. Beta strands are colored in gray and α-helices in red. The positions of the DBD and IAD are indicated.

In the absence of crystal structures of the full-length KSHV vIRFs, we have only limited knowledge about the structural similarity of the vIRFs to each other and to their cellular counterparts. One of the human IRFs that displays the most similarity to the KSHV vIRFs based on the overall amino acid sequence is human IRF4 (hIRF4), although the sequence homology is still low [13]. While viral and cellular proteins can be very distinct from each other at the amino acid level, their tertiary structures may still be conserved. Therefore, to further characterize the similarity between vIRFs, and between hIRF4 and vIRFs, we employed AlphaFold, an artificial intelligence structure prediction program, to generate the full-length structures of the vIRF1–4 proteins and compared them with each other and hIRF4. Based on this analysis, two distinct domains composed of α-helices and β-strands can be observed in each vIRF, similarly to hIRF4, which correspond to the known N-terminal DNA-binding domain (DBD) and the C-terminal IRF association domain (IAD), which are connected by an intrinsically disordered linker region with variable lengths (Figure 1). In vIRF1, the N-terminus prior to the DBD displayed high disorder, indicating high flexibility (Figure 1A). The remainder of vIRF1 had mostly ordered regions, including the DBD, a short linker (~60 aa) and the IAD at the C-terminus. In contrast, the architecture of the vIRF2–4 proteins differs from that of vIRF1 (Figure 1B–D). Their DBDs are located directly at the N-terminus, with a highly disordered, longer linker region of variable length (285–617 aa) between the DBD and the IAD. The hIRF4 protein has a short, disordered region at the N-terminus (22 aa) and C-terminus (27 aa), while its DBD and IAD domains are separated by 79 aa, possessing an intermediate level of disorder (Figure 1E). Taken together, these data suggest that hIRF4 shares structural homology with the vIRFs. Additionally, the AlphaFold prediction also indicates that the region between the DBD and the IAD domain in vIRFs is likely to be flexible, allowing some movement between the two domains, which may aid in regulating the biological activity of the vIRFs.

**Figure 2:**
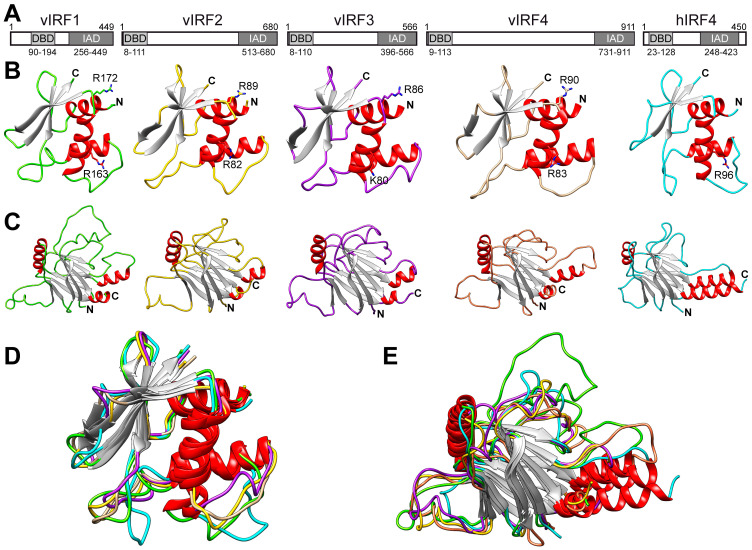
Structural comparison of the DBD and the IAD of vIRFs and hIRF4. (**A**) Schematics of KSHV vIRFs and hIRF4 indicating the DBD and IAD domains and their amino acid positions. (**B**) The ribbon diagrams of the DBDs in the vIRF and hIRF4 proteins are displayed in the same orientation. Beta strands are colored in gray and α-helices in red. The positions of arginine (R) residues 163 and 172 in vIRF1 implicated in vIRF1’s DNA binding and the basic amino acids in the equivalent position in the other vIRFs and hIRF4 are indicated. (**C**) The ribbon diagrams of the IADs in the vIRF and hIRF4 proteins are shown. The structural superposition of the DBDs (**D**) and IADs (**E**) is displayed.

Given the highly structured nature of the DBD and IAD, we further analyzed these domains separately in more detail (Figure 2). The superposition of these isolated domains revealed that while the DBDs of vIRFs and hIRF4 show low amino acid sequence identities (15–31%) (Figure 2A,B and Table 2), they are structurally more similar (58–88%) (Figure 2B and Table 2). A previous study implied that two arginine residues in the DBD of vIRF1 are involved in DNA binding, but this has not yet been confirmed experimentally [23]. We found that the location of arginine 163 in vIRF1, which interacts with DNA, is conserved in vIRF2 (R82), vIRF4 (R83) and hIRF4 (R96) (Figure 2) [23]. Importantly, these arginine residues have also been implicated in DNA binding, and, in the case of vIRF2 and vIRF4, it was shown that their mutation abolished the transcriptional activity of vIRF2 and vIRF4 [24,36,37]. In addition, our analysis revealed that arginine 172 in vIRF1, which may also be able to contribute to DNA binding, is also conserved in all vIRFs but not in hIRF4 (Figure 2B) [23]. Similarly to the DBDs, the IADs in vIRFs and hIRF4 show also low amino acid sequence identity (14–25%) but higher structural homology (61–82%) (Figure 2C and Table 2). The predicted structures indicate that the cores of the IAD domains are composed of two β-sheets and three α-helices (Figure 2C). The observation that both the DBD and IAD structures of the vIRFs and hIRF4 are highly superimposable supports the current notion that the vIRFs might have originated from cellular IRFs (Figure 2D,E).

### 3.2. Identification of KSHV Gene Promoters Regulated by vIRFs

**Figure 3:**
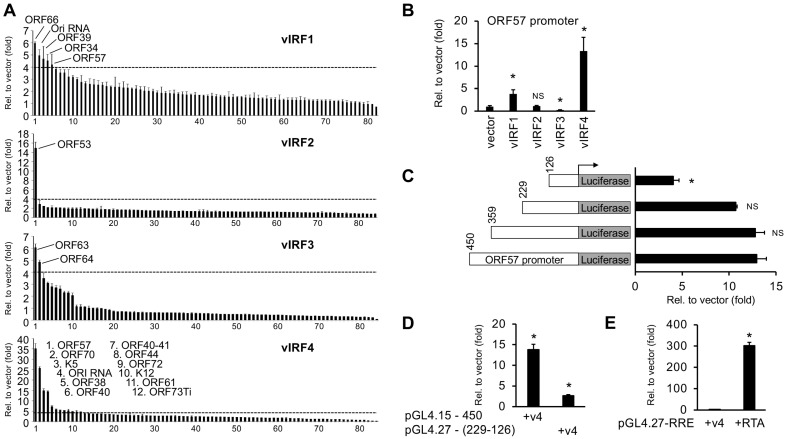
Identification of vIRF-regulated viral promoters in KSHV. (**A**) KSHV gene promoter screen with vIRFs using luciferase reporter assay. (**B**) Comparing the effects of vIRFs on the promoter activity of the KSHV ORF57 gene. The *t* tests were performed between the vector- and vIRF-transfected cells. (**C**) Measuring the induction of ORF57 promoter fragments by vIRF4. The *t* tests were calculated by comparing the promoter activity of the ORF57 promoter deletion mutants to the 450-bp full promoter of ORF57. (**D**) Testing the inducibility of the minimal promoter of the pGL4.27 reporter plasmid by vIRF4 to determine whether it can be increased via the 229–126-bp region of the ORF57 promoter. pGL4.15 containing the full ORF57 promoter was used as a positive control. The *t* tests were performed relative to the vector-transfected samples. (**E**) Luciferase reporter assay testing whether vIRF4 can induce the minimal promoter of pGL4.27 luciferase reporter plasmid via the RTA response element (RRE). RTA co-transfection was used a positive control. The *t* tests were performed relative to pGL4.27-RRE and vector co-transfection samples. The statistical significance is indicated as * *p* ≤ 0.05, and NS refers to statistically non-significant results (samples of *n* = 3).

While previous studies have shown that vIRFs can contribute to promoting the KSHV lytic cycle by inducing viral promoters, only a few KSHV gene promoters regulated by vIRFs are known so far [25,36]. To identify the KSHV gene promoters regulated by vIRFs, we conducted a series of luciferase reporter assays to screen all of the putative KSHV gene promoters with each vIRF. For this, we used a KSHV promoter reporter library, which was originally developed by Izumiya and colleagues and has been successfully used to identify all of the viral promoters regulated by the viral transcription factors RTA and K8 [32]. We co-transfected 293T cells with each of the 3xFLAG-vIRFs along with each of the 84 KSHV promoter reporter plasmids individually and measured the luciferase activity 48 h after transfection. The outcome of the promoter reporter screen is shown in Figure 3A and Table S1. We used a four-fold threshold to identify the viral promoters whose activity was the most robustly changed by vIRFs. However, we do not exclude the possibility that viral promoters whose activity is altered by vIRFs by less than four-fold may also be targets of vIRFs. Our results show that vIRF1 induced the promoters of five KSHV genes (ORFs 34, 39, 57, 66 and Ori RNA), vIRF2 could induce the promoter of ORF53, and vIRF3 increased the promoter activity of two viral genes (ORFs 63 and 64) and reduced the promoter activity of the viral gene encoding for the polyadenylated nuclear RNA (PAN) by more than 10-fold, while vIRF4 could induce the promoters of 12 different KSHV genes (Ori RNA, ORFs 70, K5, 38, 40, 40–41, 44, 57, 61, 72, K12, 73Ti).

We selected the ORF57 promoter for further analysis since it could be induced by two vIRFs, vIRF1 and vIRF4 (Figure 3B). The ORF57 promoter was defined as a 450-base-pair (bp) DNA sequence upstream of the ATG start codon of the ORF57 gene based on previous studies [38,39]. Of the two vIRFs, vIRF4 could induce the ORF57 promoter the most, which seemed to be primarily mediated by a 123-bp DNA region of the ORF57 promoter (between −229 and −126 bp), because its deletion resulted in the largest reduction in vIRF4-mediated promoter activation (Figure 3C). Consequently, we tested whether the DNA sequence between −229 and −126 bp contained a vIRF4 response element by cloning this DNA region in front of a TATA-box minimal promoter in the pGL4.27 luciferase reporter plasmid and performed a luciferase reporter assay. We found that vIRF4 only slightly induced the pGL4.27-229-126 reporter vector (2.6-fold), while the full-length ORF57 promoter in the pGL4.15-450 reporter vector was induced nearly 15-fold (Figure 3D). Since the first 126 bp of the ORF57 promoter, which includes an RTA response element (RRE), could still be induced four-fold by vIRF4 (Figure 3C), we also tested whether RRE could also mediate the effect of vIRF4 on the ORF57 promoter (Figure 3E). However, the results showed that while RTA could robustly induce the activity of the minimal promoter in pGL4.27-RRE, vIRF4 could not induce it (Figure 3E). In summary, these luciferase reporter assays show that the different vIRFs induce a limited number of distinct viral promoters, indicating a specialized role of individual vIRFs in viral gene regulation. We also found that, of the vIRFs, vIRF4 showed the strongest induction on the ORF57 promoter, and its promoter activation is likely mediated by several regions of the ORF57 promoter but not the RRE alone.

### 3.3. Identification of Host Genes Regulated by vIRFs in Primary Oral Epithelial Cells

**Figure 4:**
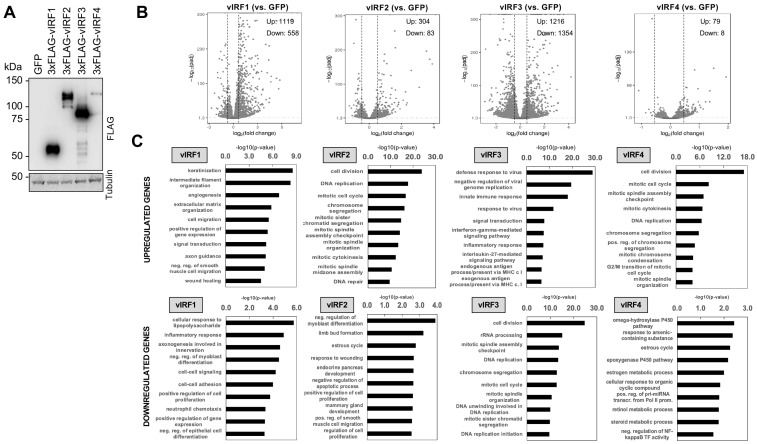
Identification of host genes regulated by vIRFs in primary oral epithelial cells. (**A**) Immunoblot analysis of the expression of vIRFs in lenti-3xFLAG-vIRF-transduced HGEP cells. (**B**) Volcano plot of the differentially expressed host genes between HGEP cells expressing GFP (control) and each of the vIRFs. (**C**) Gene ontology analysis of vIRF-regulated host genes.

Although vIRFs have been shown to be able to act as viral transcription factors, there are still no comparative transcriptomic data about the target genes of vIRFs that could reveal whether they have divergent transcriptional roles in viral infection. Additionally, the role of the vIRFs in oral epithelial cells remains unexplored. To determine which cellular genes are regulated by the KSHV vIRFs, we infected primary human gingival epithelial (HGEP) cells with lentiviruses that expressed GFP (control) or one of the N-terminally 3xFLAG-tagged vIRFs and then collected the cells at 48 h post-transduction for RNA-seq. Immunoblot analysis showed that the expression of the vIRFs was comparable, except that of 3xFLAG-vIRF4, which had low expression, similar to what was previously detected during lytic reactivation in KSHV-infected cells (Figure 4A) [17]. To analyze the RNA-seq data, we first determined the differentially expressed host genes between lenti-GFP and each of the lenti-vIRF-transduced HGEP cells. The transcriptomic analysis was based on three biological replicates, and the vIRF-regulated genes were identified based on their fold change ≥ 1.5-fold and FDR < 0.05 (Figure 4B and Table S2). Strikingly, vIRF1 and vIRF3 had the most robust effects on host gene expression. Gene ontology analysis showed that the group of vIRF1’s upregulated genes (1119 genes) was enriched in genes that encode regulators of epithelial cell differentiation, keratinization (such as TGM1, LCE3D, LCE3E, IVL) and cell migration. Based on the cellular component analysis, many of the vIRF1-induced host genes encode plasma membrane, extracellular matrix and cytoskeleton factors (Figure 4C and Table S3). Regarding vIRF3, we found that it upregulated 1216 genes, many of which are related to the regulation of the defense response to viruses or various immune response signaling pathways, such as type I and III IFNs, the inflammatory response and different chemokine and cytokine signaling pathways (Figure 4B,C). The gene ontology analysis of the 558 host genes inhibited by vIRF1 revealed that many of them are involved in the inflammatory response, the regulation of the cellular response to LPS, chemokine-mediated signaling pathways and apoptosis (Figure 4B,C and Tables S2 and S3). The vIRF3-inhibited 1354 genes are mostly related to the regulation of DNA replication, the cell cycle, rRNA processing and ribosome biogenesis and they mainly encode nuclear and cytoplasmic components (Figure 4B,C and Tables S2 and S3). Compared to vIRF1 and vIRF3, fewer host genes (304 and 79, respectively) were induced by vIRF2 and vIRF4, which can also be associated with their lower expression (Figure 4B and Table S3). Their target genes are strikingly similar to each other in terms of their biological functions, which include the regulation of DNA replication, cell division and the cell cycle (Figure 4C), and they mainly encode for nuclear proteins (Table S3). 

**Figure 5:**
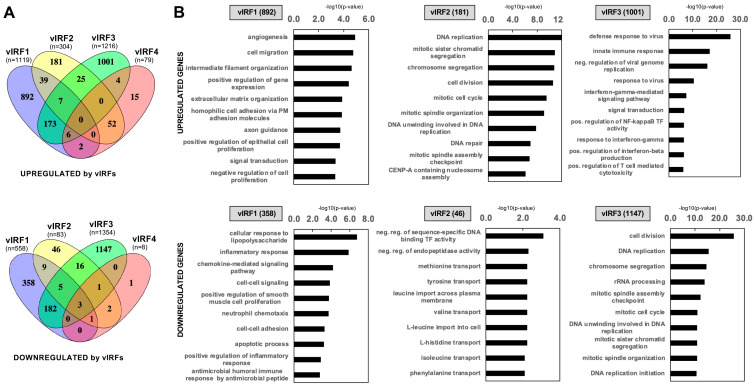
Analysis of host genes co-regulated by vIRFs in primary oral epithelial cells. (**A**) Venn diagrams showing the shared and unique upregulated and downregulated target genes of vIRFs. (**B**) Gene ontology analysis of the unique target genes of each vIRF. Note: vIRF4 is not included due to its small number of target genes.

To uncover the common and unique host target genes of vIRFs and their related biological functions, we compared the upregulated and downregulated genes of the vIRFs (Figure 5). We found that the majority of the genes that are regulated by vIRF1, vIRF2 or vIRF3 are unique, but there is a large group of genes that can be regulated by both vIRF1 and vIRF3, and most of vIRF4’s induced target genes are shared by vIRF2 (Figure 5A and Table S4 for further information). Interestingly, 52% of the genes that are induced by both vIRF1 and vIRF3 encode proteins that function by associating with the plasma membrane and the extracellular matrix (Figure 5B). Many of the genes that were inhibited by only vIRF1 but not by any other vIRFs play a role in cytokine–cytokine receptor interaction (e.g., IL1β, IL-6, CXCL3, CXCL8), which is in agreement with the known inhibitory function of vIRF1 on several immune response pathways (Figure 5B). Note that vIRF4’s GO analysis results can be found in Table S4 but are not shown in Figure 5B because no significant GO terms were identified. Importantly, we found no human genes that were induced by all four vIRFs and only three human genes (CYP1A1, EEF1A1, HECW2) were repressed by all vIRFs. Furthermore, largely distinct sets of gene ontology terms are associated with the host genes that are regulated by only vIRF1, vIRF2 or vIRF3, indicating that each vIRF regulates distinct sets of target genes in oral epithelial cells. 

**Figure 6:**
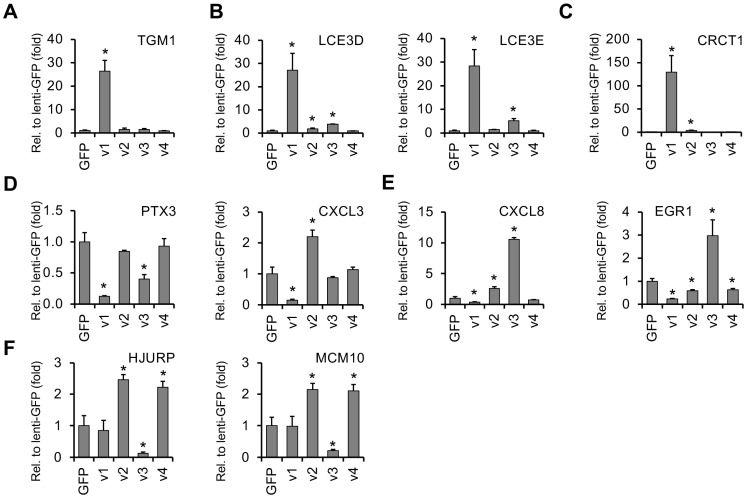
RT-qPCR confirmation and measurement of the expression of vIRF-regulated host genes in HGEP cells. (**A**) vIRF1-regulated genes. (**B**) Epidermal differentiation complex-related genes induced by vIRF1 and vIRF3. (**C**) vIRF1- and vIRF2-induced genes. (**D**) Host genes inhibited by vIRF1. (**E**) Host genes that are inhibited by vIRF1 and induced by vIRF3. (**F**) Host genes that are induced by vIRF2 and vIRF4 and repressed by vIRF3. The *t* tests were performed between lenti-GFP and the indicated lenti-vIRF samples. The statistical significance is indicated as * *p* ≤ 0.05 (samples of *n* = 3).

We confirmed the RNA-seq data by using independent lenti-vIRF transduction of HGEP and TIGK oral epithelial cells followed by RT-qPCR analysis (Figure 6). In agreement with the RNA-seq data, while the gene Transglutaminase 1 (TGM1), which plays a role in keratinization, was greatly induced by vIRF1 (Figure 6A), other epithelial development-related genes, such as LCE3D and LCE3E, were upregulated by vIRF1 and slightly by vIRF3 (Figure 6B). CRCT1 (aka NICE-1) is another member of the human epidermal differentiation complex (EDC) genes [40] and was also upregulated by two vIRFs, vIRF1 and vIRF2 (Figure 6C). Among the vIRF-repressed host genes, we confirmed that PTX3 and CXCL3 were downregulated by vIRF1 (Figure 6D), while the expression of CXCL8 (also known as IL-8) and EGR1 was reduced by vIRF1 but increased by vIRF3 (Figure 6E). Key factors in the regulation of host genomic DNA replication were also differentially affected by vIRFs. Namely, the expression of HJURP and MCM10 was reduced by vIRF3, while vIRF2 and vIRF4 induced their expression (Figure 6F).

**Figure 7:**
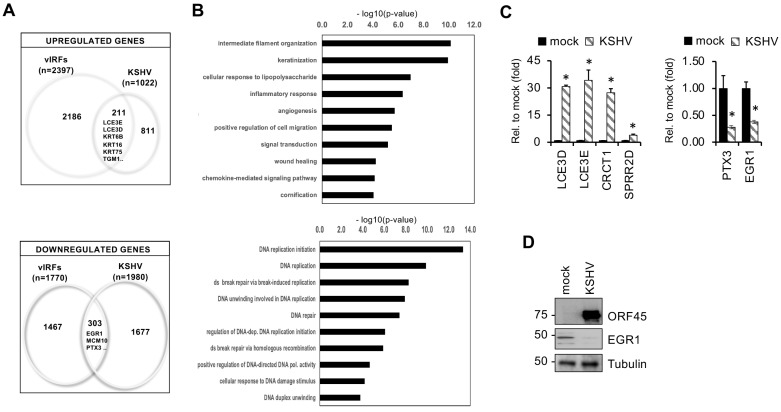
Comparison of host genes regulated by vIRFs with genes dysregulated upon KSHV infection in oral epithelial cells. (**A**) Venn diagrams showing the common and unique host genes that are regulated by the lenti-vIRFs versus during KSHV infection of HGEP cells. (**B**) Gene ontology analysis of host genes whose expression is regulated by both vIRF expression and KSHV infection. (**C**) RT-qPCR analysis of select vIRF1-regulated host genes in HGEP cells following KSHV infection at 24 hpi. Student’s *t* tests were performed between the mock and KSHV-infected HGEP samples. The statistical significance is indicated as * *p* ≤ 0.05 (samples of *n* = 3). (**D**) Immunoblot detection of a viral protein (ORF45) and a vIRF1-regulated host factor in mock and KSHV-infected HGEP cells at 24 hpi.

When we compared the vIRF-regulated genes with our previously published transcriptomic data derived from KSHV-infected HGEP cells [11], we found that 20% of the host genes that were upregulated (e.g., LCE3E, LCE3D, TGM1, etc.) and 15% of the genes that were downregulated (e.g., EGR1, MCM10, PTX3, etc.) by KSHV infection in oral epithelial cells were also similarly altered by the expression of vIRFs in KSHV-free cells (Figure 7). Gene ontology analysis showed that many of the genes induced both by vIRFs and during KSHV infection in oral epithelial cells are related to biological processes such as keratinization, epithelial cell development, the regulation of immune response pathways or cell migration. In contrast, host genes encoding factors involved in DNA replication and repair can be potential targets of vIRFs for downregulation during KSHV infection (Figure 7A,B and Table S5). Using independent RT-qPCR analysis, we confirmed that the EDC-associated genes that were induced by the vIRFs (e.g., LCE3D, LCE3E, CRCT1, SPRR2D) were also induced by KSHV infection, while the vIRF1-inhibited host genes regulating immune responses, such as PTX3 and EGR1, were downregulated during KSHV infection in HGEP cells (Figure 7C,D). We found that many of the vIRF-regulated host genes were also altered during KSHV infection in oral epithelial cells, suggesting that they can be regulated by vIRFs during KSHV infection, but this requires further investigation. In summary, we found that, despite their high structural homology to each other, each of the vIRFs regulates the expression of mostly distinct sets of host genes, indicating that each vIRF possesses specialized transcriptional functions.

### 3.4. Both the DNA-Binding Domain and the C-Terminus of vIRF1 Are Required for Its Gene Regulatory Function

**Figure 8:**
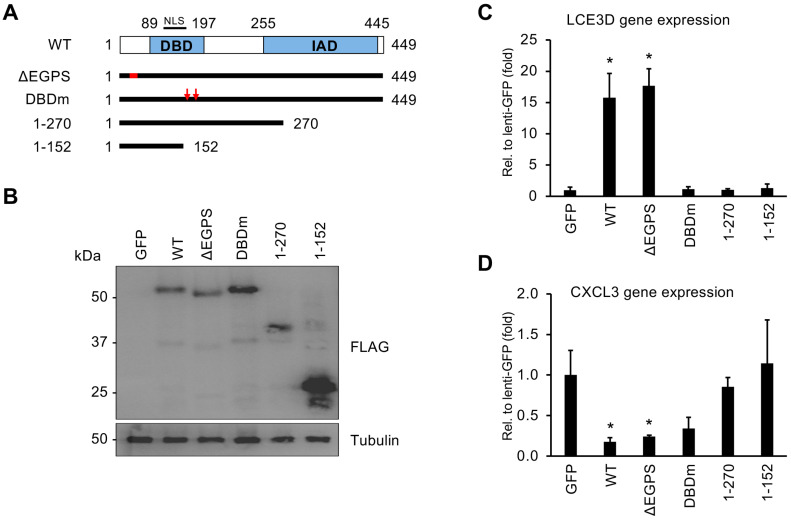
Analyzing the effect of functional mutations in vIRF1 on vIRF1-mediated host gene regulation. (**A**) Schematic representation of the wild type (WT) and mutants of vIRF1. Red arrows at DBDm indicate the R163A and R172A mutations. Red box in the ΔEGPS mutant marks the internal deletion that has been shown to abrogate the binding of vIRF1 to USP7. (**B**) Immunoblot analysis of the expression of WT and mutants of 3xFLAG-vIRF1. (**C**) RT-qPCR analysis of the effect of vIRF1 mutations on the expression of vIRF1-inducible host gene LCE3D in HGEP cells. (**D**) RT-qPCR measurement of the effect of vIRF1 mutations on the expression of vIRF1-inhibited host gene CXCL3 in HGEP cells. Student’s *t* tests were performed between lenti-GFP and lenti-vIRF1 samples. The statistical significance is indicated as * *p* ≤ 0.05 (samples of *n* = 3).

**Figure 9:**
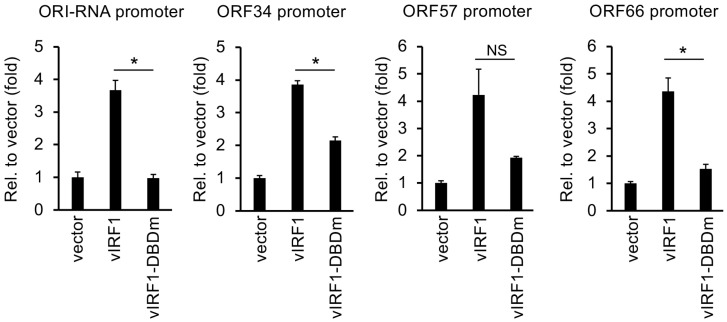
DNA-binding activity of vIRF1 is needed to induce viral promoters by vIRF1. Luciferase reporter assay was performed with the indicated KSHV gene promoter luciferase reporter plasmids and WT or DBDm of vIRF1. Student’s *t* tests were performed between WT vIRF1- and vIRF1_DBDm-transfected cells. The statistical significance is indicated as * *p* ≤ 0.05 (samples of *n* = 3).

Of the vIRFs, vIRF1 has been shown to be expressed both during latency and the viral lytic cycle in many different cell types infected by KSHV, and it is also the most rapidly and highly expressed vIRF during lytic reactivation, indicating its importance in the regulation of the KSHV life cycle [17,20]. Based on our observation that vIRF1 expression can both activate and repress host and viral genes (Figure 3 and Figure 4), we sought to determine which parts of vIRF1 are necessary for its gene regulatory function. We created four mutants of 3xFLAG-vIRF1 that were defective in different biological functions: two C-terminal deletion mutants, a DBD mutant (DBDm) created by altering arginine residues 163 and 172 to alanine and a USP7-binding mutant (ΔEGPS) of 3xFLAG-vIRF1 (Figure 8A) [23,31,41]. Immunoblot analysis showed that the expression of the vIRF1 mutants was comparable to that of WT 3xFLAG-vIRF1 (Figure 8B). Next, we tested the effect of mutations in vIRF1 on vIRF1-mediated host gene regulation by expressing vIRF1 in HGEP cells using lentiviral transduction. We found that both the DBD and C-terminus were required for the vIRF1-induced expression of LCE3D (Figure 8C), while the C-terminus was needed for the vIRF1-mediated repression of CXCL3 (Figure 8D). Using a luciferase reporter assay, we also determined that the DBD of vIRF1 is required for the vIRF1-mediated induction of its viral target promoters, such as KSHV ORI-RNA and the ORF34, ORF57 and ORF66 promoters (Figure 9). Collectively, these data indicate that the DBD of vIRF1 is essential for the upregulation of host genes and vIRF1-mediated viral promoter activation.

### 3.5. Mutation in the DBD of vIRF1 Compromises vIRF1-Mediated Gene Regulation Genome-Wide

**Figure 10:**
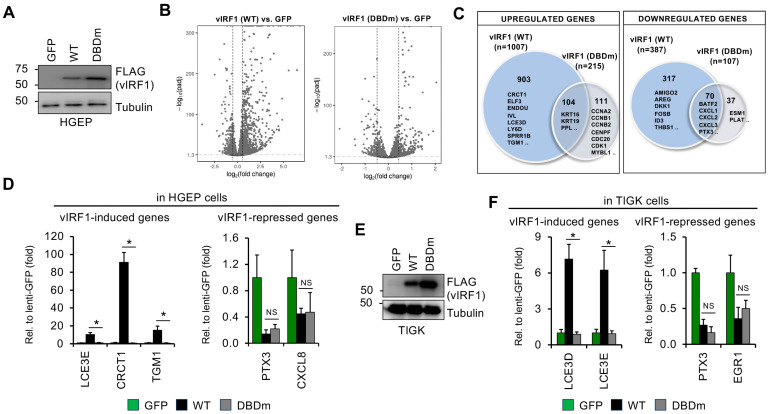
Comparing host gene expression changes between HGEP cells expressing WT vIRF1 and vIRF1 DBDm using RNA-seq analysis. (**A**) Immunoblot analysis of HGEP cells transduced with lenti-3xFLAG-vIRF1 WT or DBDm. (**B**) Volcano plot displaying the fold change in host gene expression between HGEP cells expressing GFP (control) or 3xFLAG-vIRF1 (WT or DBDm). (**C**) Venn diagram representation of the significantly upregulated or downregulated host genes that are differentially expressed between WT vIRF1 and DBDm vIRF1. Examples of genes for each category are shown. (**D**) RT-qPCR confirmation of differential expression of host genes by WT vs. DBDm vIRF1 in HGEP cells. Student’s *t* tests were performed between WT vIRF1- and vIRF1_DBDm-transduced cells. The statistical significance is indicated as * *p* ≤ 0.05 (samples of *n* = 3). (**E**) Immunoblot test of vIRF1 expression in lentivirus-transduced TIGK cells. (**F**) RT-qPCR confirmation of differential expression of host genes by WT vs. DBDm vIRF1 in TIGK cells. Student’s *t* tests were performed between WT vIRF1- and vIRF1_DBDm-transduced cells. The statistical significance is indicated as * *p* ≤ 0.05 (samples of *n* = 3).

To determine to what extent the DBD mutations affect the role of vIRF1 in the regulation of host genes, we performed a comparative RNA-seq analysis between HGEP cells expressing GFP (as a control) and the WT vIRF1 or the DBD mutant of vIRF1 (DBDm) (Figure 10 and Table S6). Immunoblot analysis confirmed the comparable expression of WT and DBDm of 3xFLAG-vIRF1 (Figure 10A). RNA-seq analysis was performed using three biological replicates and the differential gene expression was determined by applying 1.5-fold and FDR < 0.05 cut-off thresholds. We found that the DBD mutation of vIRF1 largely abrogated both its gene activator and repressor functions (Figure 10B,C). Notably, vIRF1 DBDm failed to increase the expression of 903 of the 1007 vIRF1-induced genes, many of which encode proteins associated with cell–cell adhesion, cell attachment and extracellular matrix organization based on their gene ontology analysis. This shows that the expression of these vIRF1 target genes requires the DNA-binding activity of vIRF1. Although the DBDm was able to induce the expression of a subset of genes involved in epithelial cell differentiation (e.g., KRT16, KRT19, PPL), their induction was less efficient compared to WT vIRF1, indicating that the DNA-binding activity of vIRF1 is indispensable for the robust upregulation of most of its target genes. Interestingly, we found 111 genes whose expression was only increased by the DBDm (Figure 10C). Based on their biological functions, these genes are associated with the regulation of cell division (e.g., CCNA2, CCNB2, CDC20), mitosis and chromosome segregation (e.g., CENPE, CENPF) (Table S7). These are biological functions that are not typically controlled by WT vIRF1, as shown in our earlier analysis (Figure 4). This finding suggests that the DBD mutations can alter the gene regulatory function of vIRF1.

Similarly, we found that most of the host genes downregulated by WT vIRF1 were not downregulated by the DBD mutant vIRF1 (Figure 10C). Our analysis showed that most of the repressed vIRF1 gene targets, such as those that play a role in cytokine and inflammatory responses (e.g., IL1B, TNFSF4, NODAL), IFN-gamma production or apoptosis, were all downregulated only by WT vIRF1 and not by DBDm, indicating that the DNA-binding activity of vIRF1 is also required for the repression of its target host genes (Figure 10C and Table S7). However, a small subset of genes related to the inflammatory response and chemokine signaling (e.g., PTX3, CXCL1, CXCL3) or the defense response to viruses were still repressed by the DBD mutant (Figure 10C and Table S7). These RNA-seq results were further confirmed in independent experiments in both HGEP and TIGK cells (Figure 8C,D and Figure 10D–F). We note that although TGM1 is one of the most strongly induced genes by vIRF1 in HGEP, it was not induced by vIRF1 in TIGK cells (Figure 10D), suggesting that cell-type-specific differences in vIRF1-mediated gene regulation can exist. Altogether, these results demonstrate that the DNA-binding domain of vIRF1 is generally required for vIRF1-mediated gene regulation. However, we also found that there is a unique group of host genes (such as the inflammatory response genes) whose regulation by vIRF1 does not depend on its DNA-binding activity. We speculate that they might be controlled by vIRF1 through modulating cell signaling pathways, as shown by others [21].

### 3.6. DNA-Binding Domain of vIRF1 Is Necessary to Promote Accumulation of Activating Histone Modifications on Its Target Gene

**Figure 11:**
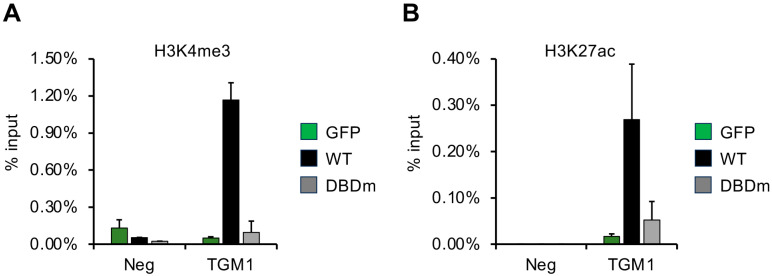
vIRF1 increases the level of activating histone modifications on the TGM1 promoter. WT or DBDm of vIRF1 was expressed in HGEP cells for 2 days followed by ChIP analysis to test the accumulation of (**A**) H3K4me3 and (**B**) H3K27ac on the TGM1 promoter (0.7 kb upstream of the transcription start site of the TGM1 gene).

Transcription factors are known to exert their activities on their target promoters through altering the level of histone modifications. We hypothesized that vIRF1 may control the expression of its target genes by regulating the chromatin modifications on the gene regulatory elements of its target genes. To investigate the effect of vIRF1 and uncover the contribution of its DNA-binding domain, we performed a ChIP assay to test the level of the activating histone marks H3K4me3 and H3K27ac on the promoter of the vIRF1-induced gene TGM1. An intergenic region of the host genome (Neg) was used as a negative control (Figure 11). HGEP cells were transduced with lentiviruses expressing GFP (control), 3xFLAG-vIRF1 (WT) or the DNA-binding domain mutant of 3xFLAG-vIRF1 (DBDm) and the ChIP assay was conducted 48 h after the lentiviral transduction. We found that WT vIRF1 greatly increased the enrichment of H3K4me3 (Figure 11A) and H3K27ac (Figure 11B) on the TGM1 promoter, while DBDm could not. These results correlate with WT vIRF1, but not the DBDm, being able to induce TGM1 expression. These data suggest that the DNA binding of vIRF1 is crucial for its ability to increase the level of activating histone marks, which can lead to the increased expression of its target genes in oral epithelial cells.

## 4. Discussion

It is still largely unknown how the viral factors of KSHV modulate cell signaling pathways and host genes in oral epithelial cells to facilitate lytic viral infection in the oral cavity, which is a major site of primary KSHV infection in humans. While previous studies have extensively investigated the immunomodulatory functions of vIRFs, their role in gene regulation, which could contribute to promoting the lytic KSHV infection of oral epithelial cells, remains understudied [21]. In this study, we provided a comparative analysis of the protein structure and the gene regulation potential of the four vIRFs expressed by KSHV. Based on the structure prediction, we concluded that despite their low amino acid sequence similarity, the vIRFs share high structural homology within the DBD and IAD domains, which are similar to those of hIRF4. Although the vIRFs share structural similarity in the DBD and IAD regions, we demonstrated that there is functional divergence between the vIRFs, as each vIRF induces different viral promoters and regulates distinct host genes in oral epithelial cells. Most notably, even though the individual vIRFs regulate hundreds of human genes in oral epithelial cells, there are almost no genes that are regulated by more than two vIRFs, suggesting a highly specialized non-overlapping transcriptional function for the vIRFs. This finding indicates that despite their significant structural identity, each vIRF has evolved to possess different transcriptional functions. In addition, we also provided evidence for the requirement of the vIRF1 DBD for its gene regulatory function genome-wide and its ability to alter the levels of histone modifications on an induced target gene (TGM1). Our findings support the notion that KSHV vIRFs can function as viral transcription factors controlling the expression of specific viral and host genes that can play a role in promoting primary KSHV infection in oral epithelial cells.

The cellular IRFs possess two domains, an N-terminal DBD and a C-terminal IAD, which are connected by a flexible linker region. The DBDs of IRFs contain several structurally critical tryptophan amino acids, enabling them to form a helix–turn–helix domain, which binds to DNA. The IAD mediates many of the IRF protein–protein interactions, including IRF homo- and heterodimerization and binding to multiple different transcription factors (TFs). While the crystal structure of the DBDs of vIRF1 and vIRF2 has been solved [23,24], the tertiary structures of full-length vIRFs have not. The AlphaFold analysis revealed that the vIRFs share apparent structural homology with each other and with hIRF4. They all possess an N-terminal DBD and a C-terminal IAD structurally ordered domain composed of α-helices and β-strands, which are connected by an intrinsically disordered linker region of variable lengths. The linker region in cellular IRFs is flexible, allowing potential intramolecular interactions between the DBD and IAD, which can influence the DNA-binding activity of IRFs. Given the high structural similarity between cellular hIRF4 and vIRFs, it is possible that the DNA-binding activity of vIRFs is regulated by a similar intracellular interaction. This could explain the strong interaction between DNA and the vIRF1 DBD alone compared to the full-length form of vIRF1 in vitro. Interestingly, although the DBDs of KSHV vIRFs lack the structurally critical tryptophan residues essential for the DNA-binding activity of cellular IRFs, both vIRF1 and vIRF2 have been shown to bind to DNA in vitro, suggesting that the DNA-binding mechanism of vIRFs and cellular IRFs is likely different. However, it remains to be determined to what extent the DNA binding of each of the vIRFs is crucial in regulating the expression of their host or viral target genes.

Previous ChIP-seq studies mapped the binding sites of vIRF2, vIRF3 and vIRF4 on the human genome in different cell lines, revealing that, in many cases, they can overlap with cellular IRF-binding sites [24,42,43]. As a matter of fact, vIRF3 has been shown to co-occupy many of the genomic sites that IRF4 binds to in PEL cells to promote the expression of key survival genes of PELs [43]. However, there is still no firm evidence for KSHV vIRFs being able to directly bind to the same DNA sequences as IRFs. Cellular IRFs are known to regulate many different cellular processes, such as the immune response, cell cycle and development. The RNA-seq analysis of the vIRF gene targets in this study shows that vIRFs can also control genes related to many of the cellular IRF-regulated biological processes. Furthermore, we found that the biological processes regulated by the vIRFs were distinct and even opposing in some cases. For example, while vIRF2 and vIRF4 induce genes related to the regulation of cell division, DNA replication and cell cycle progression, vIRF3 inhibits these processes. A similar conclusion can be made in relation to the immune pathways, with vIRF1 inhibiting host pathogen defense responses and vIRF3 activating defense responses to viruses and the innate immune response. This is exemplified by the observation that vIRF1 induced while vIRF3 repressed CXCL3 and CXCL8 gene expression. While the innate immune-blocking activity of vIRF1 is well known, this is the first study to show that vIRF3 may have a divergent function in oral epithelial cells. In other cell types, vIRF3 has been shown to bind IRF7 to inhibit its DNA-binding activity [44]; however, the oral cavity is a unique body site in that it permits lytic viral gene expression. It is possible that vIRF1 and vIRF3 counteract each other in the oral epithelium to allow for a low level of viral replication, which would create a unique niche for chronic KSHV infection and viral shedding.

Among the most strongly induced genes, based on our RNA-seq analysis, were the vIRF1-induced clusters related to epidermal differentiation. Previous transcriptome analyses of KSHV-infected oral epithelial cells in our labs [11] and by the Jung lab using an oral epithelial air–liquid interface model [45] revealed that KSHV infection induced genes related to keratinization and cell–cell adhesion, such as TGM1, which we identified here to be induced by vIRF1 in oral epithelial cells. The expression of epidermal differentiation markers in the superficial layers of the oral epithelium has been shown to induce KSHV lytic replication in organotypic raft cultures and organoids [45,46,47], suggesting that vIRF1 may promote the viral lytic cycle in the oral epithelium. A previous single-cell RNA-seq experiment demonstrated that KSHV infection of the basal oral epithelial layer led to the enrichment of host signaling pathways related to cell adhesion and extracellular matrix organization [45], which were pathways that were inhibited and induced by vIRF1, respectively, in our study. Further studies are needed to uncover the role of vIRFs in KSHV infection of oral epithelial cells and to determine whether other viral factors may mask independent functions of the vIRFs during primary infection. 

While the role of vIRFs has been tested in the regulation of some KSHV gene promoters, this is the first study to comprehensively contrast the effects of all four vIRFs on all viral promoters. We found that each vIRF stimulated distinct sets of promoters, with vIRF1 and vIRF4 having the most targets and with the ORF57 promoter stimulated by both factors. A previous study similarly demonstrated that vIRF4 could slightly activate the ORF57 promoter in HeLa cells, but this effect was amplified with the co-transfection of RTA [36]. Notably, this study also showed that only the full-length vIRF4 had this synergistic effect, emphasizing both the N-terminal and DBD regions for promoter stimulation [36]. Another study in 293T cells demonstrated that vIRF1 can bind the KSHV K3/vIL6 promoter region and luciferase assays demonstrated the slight vIRF1-mediated activation of the promoter [25]. The fact that vIRFs can interact with many distinct viral and host transcription factors and chromatin regulatory factors in KSHV-infected cells, which can modulate the transcriptional activity of vIRFs in different cell types, can explain why the lack of vIRFs has different effects on the KSHV lytic cycle in distinct cell types [17,42,48,49].

Previous studies have shown that the DBD of vIRF2 and vIRF4 is important for vIRF-mediated gene regulation [24,36,42]. In agreement with these studies, our results indicate that the vIRF1-mediated induction of viral promoters and host genes also requires its intact DNA-binding domain, while the suppression of cytokine gene expression is somewhat independent of the DNA binding of vIRF1. In addition, our ChIP experiments further support the critical role of vIRF1’s DBD in regulating gene transcription. The inability of the vIRF1 DBDm to promote the accumulation of the activating histone modifications H3K4me3 and H3K27ac on the TGM1 promoter supports the notion that direct DNA binding by vIRF1 is essential for its transcriptional activation function. Although the mechanism by which vIRF1 can regulate the enrichment of histone modifications at its target gene promoters in oral epithelial cells remains unknown, vIRF1 has been shown to be associated with several chromatin regulatory factors (e.g., HCFC1, CBP, USP7, JMJD1C, PRMT5) that could be utilized by vIRF1 to modulate the viral and host epigenomes [49]. The observation that certain host genes related to the inflammatory response and chemokine signaling can still be repressed by vIRF1 DBDm suggests alternative mechanisms of gene regulation that do not rely solely on direct DNA binding. Further studies are needed to reveal how vIRFs exert their genome-wide gene regulatory functions through binding to DNA, interacting with other TFs and chromatin regulatory factors and/or influencing cell signaling pathways.

In summary, our study is the first to provide a comparative genome-wide analysis of the gene regulatory functions of the structurally homologous vIRFs in oral epithelial cells. Elucidating the molecular determinants required for the transcriptional functions of vIRFs in oral KSHV infection will advance our understanding of how KSHV can modulate its host cell environment to promote viral infection. These findings will not only contribute to our knowledge of KSHV viral gene regulation but can also help in finding new avenues for the development of therapeutic strategies aimed at treating KSHV-induced pathologies.

## Figures and Tables

**Table 1:** The sequences of the primers given in 5′ to 3′ orientation.

Gene Target	Primer Sequence in 5′ to 3′ Orientation
For RT-qPCR	
TGM1_F	CAGTGAAACTGCACCTCTACCT
TGM1_R	CTCCACTTCCTTCTTGGTCTCC
LCE3D_F	ATACCCTCTTCTGGCTTTCACA
LCE3D_R	AGGTACAGGGGTAAGGGAACAT
LCE3E_F	CTTCTCCTGCCTCTCTGCAC
LCE3E_R	TTCTTTGGGGGACACTTGGG
CRCT1_F	CCTGACCCCGATGTGATTTTTC
CRCT1_R	CAAGCCCGTGTAGACAGTAGG
PTX3_F	GGGACAAGCTCTTCATCATGCT
PTX3_R	GTCGTCCGTGGCTTGCAGCA
CXCL3_F	GTCCGTGGTCACTGAACTGC
CXCL3_R	GGGGGACCTTACATTCACACT
CXCL8_F	AGCTCTGTGTGAAGGTGCAGT
CXCL8_R	TAAATTTGGGGTGGAAAGGTT
EGR1_F	CAAGAGGCATACCAAGATCCA
EGR1_R	GGACGGGTAGGAAGAGAGAGA
HJURP_F	CTGCCCAAGAGCGATTCATCT
HJURP_R	AACGTGAAGGTCAGATGTCTG
MCM10_F	ACGGCGACGGTGAATCTTAT
MCM10_R	TCAGTTGACTGTGATGCGGG
SPRR2D_F	TTATCAACAGCAGCAGTGCAAG
SPRR2D_R	GCTGTGGACACTTTGGTGATG
18S_F	TTCGAACGTCTGCCCTATCAA
18S_R	GATGTGGTAGCCGTTTCTCAGG
For ChIP-qPCR	
Neg_F	CAGGATCTCCGAGAATCAGC
Neg_R	GAGTTGGGAGAGCTGTCAGG
TGM1_F	AGGGAGATCAGAGTAGGAGCAA
TGM1_R	AAGGTACTCTGTACCCCTGGAA

**Table 2:** Amino acid sequence and structural identity between DBD or IAD domains of vIRFs and hIRF4.

**DBD**	**vIRF1**	**vIRF2**	**vIRF3**	**vIRF4**	**hIRF4**	Sequence identity (%)
vIRF1		23	20	17	22	
vIRF2	66		26	31	23	
vIRF3	73	87		25	15	
vIRF4	69	88	77		17	
hIRF4	71	60	61	58		
**IAD**	**vIRF1**	**vIRF2**	**vIRF3**	**vIRF4**	**hIRF4**	
vIRF1		22	15	15	18	
vIRF2	71		23	17	16	
vIRF3	66	82		25	17	
vIRF4	71	71	62		14	
hIRF4	61	66	68	61		
Structural identity (%)						

## Data Availability

Data are contained within the article and supplementary materials.

## Supplementary Materials

The following information can be downloaded at: https://www.mdpi.com/article/10.3390/v16060846/s1, Table S1: Summary of the promoter luciferase reporter assays with vIRFs. Table S2: Differential gene expression between lenti-GFP and lenti-vIRF transduced HGEP cells. Table S3: Gene ontology biological process and cellular component analyses of differentially expressed host genes identified between lenti-GFP and each of lenti-vIRF transduced HGEP cells. This table is related to Figure 4. Table S4: Gene ontology biological process and cellular component analyses of host genes that were co-regulated by several vIRFs or regulated by only one specific vIRF. This table is related to Figure 5. Table S5: Listing the gene ontology biological process and cellular component analyses of host genes that are dysregulated by both KSHV infection and the vIRFs. This table is related to Figure 7. Table S6: Differential gene expression between lenti-GFP and lenti-vIRF1 or lenti-vIRF1/DBDm transduced HGEP cells. This table contains an independent set of samples (lenti-GFP, lenti-vIRF1), which were processed together with lenti-vIRF1/DBDm. This table is related to Figure 10. Table S7: Gene ontology biological process analysis of host genes that were differentially expressed upon lenti-vIRF1 or lenti-vIRF1/DBDm transduction in HGEP cells compared to lenti-GFP transduced cells. This table is related to Figure 10.
